# An Overview of Biomarkers and Molecular Signatures in HCC

**DOI:** 10.3390/cancers2020809

**Published:** 2010-05-07

**Authors:** Seon-Hee Yim, Yeun-Jun Chung

**Affiliations:** 1 Integrated Research Center for Genome Polymorphism, The Catholic University of Korea School of Medicine, 505 Banpo-dong, Seocho-gu, Seoul 137-701, Korea; E-Mail: lyra@catholic.ac.kr (S.Y.); 2 Department of Microbiology, The Catholic University of Korea School of Medicine, 505 Banpo-dong, Seocho-gu, Seoul 137-701, Korea

**Keywords:** hepatocellular carcinoma, biomarker, molecular classification

## Abstract

Hepatocellular carcinoma (HCC) is the third most common cause of cancer mortality worldwide. Although most HCCs seem to originate from the accumulation of genetic abnormalities induced by various risk factors, underlying mechanisms of hepatocarcinogenesis remain unclear. Long-term survival of HCC patients is also poor, partly due to HCC recurrence. Although serum alpha-fetoprotein (AFP) level is a useful marker for the detection and monitoring of HCC, AFP levels may remain normal in the patients even with advanced HCC. To identify useful biomarkers for HCC, many studies have been conducted on molecular events such as genetic and epigenetic alterations, and gene expression. This review summarizes recent studies of potential molecular markers for diagnosis and monitoring metastasis or recurrence of HCC.

## 1. Introduction

Hepatocellular carcinoma (HCC) is the third most common cause of cancer mortality worldwide, and liver cirrhosis is the most important predisposing factor for it [1]. Hepatitis B and C viral infection is the most common underlying cause of chronic liver disease leading to liver cirrhosis, and aflatoxin B1 and alcohol are also well-known risk factors. Although most HCCs seem to be originated from the accumulation of genetic abnormalities induced by various risk factors, underlying mechanisms of hepatocarcinogenesis remain unclear.

Long-term survival of HCC patients is poor, partly due to HCC recurrence, which up to 80% of the patients experience even after curative resection [2]. Although serum alpha-fetoprotein (AFP) level is a useful marker for the detection and monitoring of HCC, AFP levels may remain normal in up to 30% of the patients with advanced HCC [3]. Various HCC markers have been suggested, but the overall performance has been unsatisfactory in terms of sensitivity and specificity. To improve HCC prognosis, it is imperative to find useful markers for early diagnosis and monitoring of recurrence of HCC.

To identify candidate biomarkers for HCC, many studies have been conducted on molecular events such as genetic and epigenetic alterations, and gene expression. High-throughput omics-based technologies have also enabled the genome-wide interrogation of these changes. This review summarizes recent studies of potential molecular markers for diagnosis and monitoring metastasis or recurrence of HCC.

## 2. Candidate Markers for Diagnosis

### 2.1. Serum DNA

It is known that some tumors release cells and cell lysis material including DNA into the blood, which could be used as potential biomarkers for detection of cancers. Zhang *et al*. reported that methylated alleles of *p16*, *p15*, and *RASSF1A* were found in serum DNA of 44%, 24%, and 70% of HCC patients, but detected in 4%, 0%, and 6% of control serum DNA samples, respectively [4]. These methylated alleles could be detected one to nine years before the clinical diagnosis of cancer. The average time to clinical diagnosis of cancer since methylated alleles were detected in serum was 4.3 years for p16, 3.4 years for p15, and 4.4 years for *RASSF1A*. Since a series of genetic and epigenetic events occur during the long period, these genes seem to be potential epigenetic biomarkers for early detection of HCC or precancerous lesions non-invasively [4]. However, they did not examine the relationship between these genes and various etiologic factors. Wong *et al*. reported a correlation between *p16* hypermethylation in HCC tissues and serum DNA and that *p16* hypermethylation was not detected in the serum of patients with other chronic liver diseases such as liver cirrhosis or hepatitis [5]. However, 17% of cirrhotic patients had been reported to have serum DNA with aberrant *p16* methylation in another study, which may reflect the lack of standardized processing of blood samples and analytic methods of serum DNA [6]. No studies on the relationship between methylation status of *p15* and *RASSF1A* in serum DNA and cirrhosis have been reported.

### 2.2. RNA Expression

RNA expression profiles are one of the most successful markers that can be used to classify many solid tumors including HCC, based on molecular characteristics. Kim *et al*. examined the mRNA expression of tissue samples derived from cirrhotic patients of various etiologies using cDNA microarray and reported a molecular signature containing 273 significantly altered genes in HCC [7]. The misregulated genes were clustered into several functional pathways, including metal transport, blood coagulation, and immune response, which can be involved in hepatocarcinogenesis. Among them, 12 genes encode secretory proteins detectable in sera, which may be used as markers for early diagnosis of HCC and as targets for chemoprevention. Misregulation of the genes in this signature is common in patients with HBV and HCV infection, hemochromatosis, and Wilson’s disease, but not very common in patients of other etiologies. This is consistent with the clinical findings that patients with cirrhosis caused by HBV infection, HCV infection, and hemochromatosis have a higher risk of developing HCC compared with those with cirrhosis caused by other factors [8].

Jia *et al*. identified five genes (*GPC3, PEG10, MDK, SERPINI1*, and *QP-C*), which were overexpressed in most of the HCC samples, including those with normal serum AFP and early stage tumors [9]. They showed that a combined use of these five genes could classify noncancerous hepatic tissues (100%) and HCC (71%). To use expression profiles as biomarkers, invasive techniques are required to get the tissues, but markers, which encode extracellular proteins or secretory proteins, can be used to develop non-invasive markers. Among the five genes, *GPC3, MDK*, and *SERPINI1* encode extracellular proteins, which can be detected in serum. They tested serum MDK and found that it can distinguish normal and cirrhotic individuals from HCC patients, including those with normal AFP and small tumors, which could be used as a diagnostic marker. Most of HCC cases from this study were positive for hepatitis B.

Budhu *et al.* reported that livers from metastatic HCC patients have a different gene expression pattern compared with the livers from patients without metastatic HCC, and suggested a 17 gene predictor of HCC venous metastasis [10]. Liver tissues bearing metastatic HCC showed a decrease of pro-inflammatory Th1-like cytokines and an increase of anti-inflammatory Th2-like cytokines. It suggests that the predominant humoral cytokine response in the liver inducing anti-inflammatory/immune-suppressive responses may play a role in promoting HCC venous metastases. The identified predictor is only applicable for operable HCC patients and patients positive for HBV due to the characteristics of the cohort.

### 2.3. Serum Protein

#### 2.3.1. AFP and AFP-mRNA

Although total AFP has been a useful marker for diagnosis and monitoring of HCC, it is often difficult to distinguish tumors from benign liver diseases based on the elevated AFP level. Recently, a hepatoma-specific AFP (HS-AFP) subfraction was reported to be superior to total AFP level in both sensitivity and specificity in differentiating benign liver diseases from malignant ones [11,12]. Total AFP can be divided into three glycoforms, AFP-L1, AFP-L2, and AFP-L3. Among them, AFP-L3 is a HS-AFP found in the sera of HCC patients. There was a relationship between AFP-L3 percentage with HCC differentiation, metastasis and relapse, suggesting that the percentage of HS-AFP may be a more specific marker than total AFP for early diagnosis of HCC and its recurrence [13,14].

AFP-mRNA from peripheral blood mononuclear cells by RT-PCR has also been studied in recent years [15]. Although AFP-mRNA is frequently detected in the blood of patients with benign liver diseases or of HCC patients without extrahepatic metastases, it seemed to identify more aggressive tumors in terms of clinical behaviors. If hepatocyte-specific mRNAs are detected in circulating blood, it could reflect the presence of circulating, presumably malignant liver cells and suggest the likelihood of hematogenous metastasis.

#### 2.3.2. Hepatoma-specific Gamma-glutamyl Transferase (GGT) Isoenzyme

GGT is an enzyme that catalyzes the degradation of glutathione and other gamma-glutamyl compounds. It is highest in embryonic livers and decreases to the lowest levels right after birth, but total GGT activities in patients with liver disease and extrahepatic tumors are high [16]. GGT is divided into several subfractions, among which the hepatoma-specific GGT bands (including I', II, and II', HS-GGT) in sera of HCC patients have been used for diagnosis of HCC. HS-GGT can only be found in sera of HCC patients, and its analysis may improve the specificity and sensitivity of HCC diagnosis [17]. Yao *et al*. showed that the circulating HS-GGT activity was elevated in 86% of the HCC patients, but in less than 3% of patients with other diseases, and that there is an increasing tendency of total RNA concentrations also observed from liver cancer to distal noncancerous tissues [16]. The serum HS-GGT level can be a sensitive tumor marker for diagnosis or differentiation of HCC.

#### 2.3.3. Transforming Growth Factor (TGF)-β1

TGF-β1 is one of TGF-β isoforms, which arrests the cell cycle in the G1 phase and elicits inhibition of cell proliferation and triggering apoptosis [18]. Although it is a growth inhibitor, the overexpression of hepatic TGF-β1 was found in HCC tissues and shown to be correlated with carcinogenesis, progression and prognosis of HCC [19,20]. TGF-β1 expression is also associated with the degree of HCC differentiation and status of HBV replication, but neither to the size nor to number of tumors [21]. However, the sensitivity and specificity of circulating TGF-β1 level as a non-invasive marker have been rarely studied in HCC.

#### 2.3.4. Insulin-like Growth Factor (IGF)-II and Related Factors

IGF-II is a mitogenic polypeptide related to insulin and thought to serve as a growth factor in various cancers through coexpressing IGF-II and IGF-I receptors, which is a kind of fetal growth factor and highly expressed during hepatocarcinogenesis [22]. It has been suggested that IGF-II may play a role in the neovascularization of HCC by increasing vascular endothelial growth factor (VEGF) [23,24]. The circulating free IGF-II levels were significantly higher in HCC patients than in those with chronic hepatitis or liver cirrhosis. The circulating IGF-II mRNA was positive in 34% of HCC patients, but negative in other liver diseases, extrahepatic tumors, and normal controls. The circulating IGF-II mRNA correlated with the stage of HCC, and was detected in 100% of HCC with extrahepatic metastasis. No significant relationship was found between tumor sizes and circulating IGF-II mRNA. The expression of free IGF-II and IGF-II mRNA may be useful markers for diagnosis of HCC and its extrahepatic metastasis, and monitoring recurrence [12].

#### 2.3.5. Hepatocyte Growth Factor (HGF)

The HGF is the most potent growth factor for hepatocytes and has been reported to be elevated in a number of liver diseases, including acute and chronic hepatitis, liver cirrhosis, HCC, primary biliary cirrhosis, and fulminant hepatic failure [25]. Stellate cells and myofibroblasts are induced to secrete HGF by tumor cell products and HGF stimulates tumor cell invasiveness in turn [26]. Vejchapipat *et al*. reported that patients with inoperable HCC had higher levels of serum HGF than healthy controls, and serum HGF was negatively correlated with survival [27]. They suggested that the elevated HGF levels in HCC patients may reflect the impaired clearance of HGF due to significant liver damage or may be caused by increased HGF production to regenerate hepatocytes [27]. Similarly, Yamagamim *et al*. found that the serum HGF was significantly higher in patients with HCC than in patients with chronic hepatitis or cirrhosis [28]. In addition, patients with relatively higher HGF concentrations had an ectopic recurrence or a diffuse infiltration of HCC. They followed up the patients after measuring the initial HGF levels and found that the cumulative incidence of HCC in patients with higher initial HGF concentrations are higher than those with lower HGF. They suggested that the elevated serum HGF is probably produced by infiltrating mesenchymal and cancer cells based on their observations; in one patient whose HGF level was below the detection limit at the initial examination, the concentrations increased over the time and exceeded 0.4 ng/mL just before HCC was detected; HGF concentrations were higher in patients with diffuse carcinomas or with multiple cancers than in patients with a single cancer [28]. It may be useful to evaluate the HGF level for the detection and follow-up of HCC.

#### 2.3.6. Heat Shock Proteins (HSPs)

HSPs are induced in cells under various stress conditions, including carcinogenesis. Enhancement of intracellular HSP is related to the formation and development of HCC and reported as a vital marker indicating the progression and aggravation of HCC [29]. The HSP gp96 expression was reported to increase as the HBV-induced disease progressed from chronic hepatitis to cirrhosis then HCC, and to be correlated with the degree of tumor differentiation and tumor size, but not with the number of tumors [30]. Sakamoto *et al*. reported the significant overexpression of HSP70 in early HCC compared with precancerous lesions, and in advanced HCC compared with early HCC. HSP70 has been shown to be negative in other benign nodular lesions, hepatocellular adenoma and focal nodular hyperplasia. Hence, HSP70 might work as a molecular marker to differentiate between benign and malignant liver nodules [31].

#### 2.3.7. Complement C3a

Complement C3a, which is produced from C3 by C3 convertase, has been reported as a potent inflammatory mediator of innate immune response, and to contribute to the early priming stages of hepatocyte regeneration after toxic injury and partial hepatectomy [32]. Lee *et al*. found that complement C3a was elevated in the sera of patients with chronic hepatitis C and HCV-related HCC, but not in those with HBV-related HCC. It supports that the molecular hepatocarcinogenesis might be different between HBV-related and HCV-related HCC, and therefore the effect on the expression levels of C3a might also be different [33].

#### 2.3.8. Glypican-3 (GPC3)

GPC3 was suggested as a possible tumor marker for HCC, since the levels of GPC3 were significantly high in the serum of HCC patients, but undetectable in healthy donors and patients with benign liver diseases [34,35]. Capurro *et al*. showed that GPC3 stimulates the growth of HCC cells by stimulating the Wnt pathway through facilitating the interaction between the Wnts and their signaling receptors [36]. GPC3 was found to be expressed in small tumors, indicating its potential as a diagnostic marker for early stage HCC. The study also showed that the combined use of GPC3 and AFP would significantly increase the sensitivity of the test without compromising specificity.

#### 2.3.9. Squamous Cell Carcinoma Antigen (SCCA)

SCCA is a member of the family of serine protease inhibitors named serpins and has been reported to be overexpressed in HCC tissues [37]. Giannelli *et al*. compared HCC and liver cirrhosis and reported that the combination of serum AFP and serum SCCA yielded a correct serologic diagnosis in 90.8% of the HCC patients [37]. Guido *et al*. reported that SCCA was poorly expressed in regenerative tissue, but strongly expressed in dysplastic nodules, suggesting a role as a potential marker for early detection of HCC [38]. It has been reported that SCCA can react with the IgM class of immunoglobulins to form the immunocomplexes SCCAIC, which is detectable in the serum of HCC patients. They suggested that SCCA-IgM ICs alone or in combination with AFP, can increase the sensitivity for diagnosing HCC significantly [39]. Tretoli *et al*. reported that serum SCCA levels in cirrhotic patients were significantly lower than in HCC patients, but that there was no significant correlation between tissue and serum levels of SCCA [40]. 

### 2.4. Immunohistochemistry (IHC) Markers

IHC plays an important role in the diagnosis of HCC. A number of IHC markers are established including several cytokeratins such as HepPar 1 and pCEA. However, none of these markers are specific enough for the diagnosis of HCC and some of them appear or disappear during tumorigenesis, which make a diagnosis difficult. Therefore, the combination of IHC markers is also used for a precise diagnosis.

#### 2.4.1. Hepatocyte Paraffin 1 (HepPar 1)

HepPar 1 has been acknowledged as the most sensitive and specific IHC marker for HCC and reflects hepatocyte differentiation [41,42]. Because it is positive in normal liver and adenomas, it is not useful for distinction of benign *versus* malignant liver lesions. HepPar 1 is more likely to be negative in poorly differentiated and sclerosing HCC (sensitivity 50% or less). Although most adenocarcinomas are negative for HepPar 1, gastric, esophageal, and lung adenocarcinomas can occasionally show strong positive reactions. Given the relatively higher frequency of metastatic cancer to the liver from these sites, the predictive value of positive HepPar 1 is not high. Rare carcinomas with hepatoid morphology occur in the gastrointestinal tract and pancreas and are positive for HepPar 1 [42].

#### 2.4.2. Polyclonal Carcinoembryonic Antigen (pCEA)

Overall efficiency of pCEA IHC for HCC in study was approximately 90% [42]. Diffuse cytoplasmic expression of pCEA is observed in various adenocarcinomas. HCC shows a canalicular pattern in 60% to 90% of cases [42,43], which is characteristic of HCC but not observed in other adenocarcinomas. Sensitivity is high for well- and moderately differentiated HCCs (~80%), but low in poorly differentiated one (25%–50%). In several studies, the frequency of biliary canalicular stain decreased with grade; 58%–100% well-differentiated, 25%–80% moderately differentiated, and 0%–73% poorly differentiated HCC expressing the antigen [42].

#### 2.4.3. Glypican 3 (GPC-3)

GPC-3 is an oncofetal protein, as it is normally expressed in fetal liver and placenta but not in normal adult liver. GPC-3 is reported to be expressed in 64% to 90% of HCCs, but not in benign masses, which helps to distinguish benign *versus* malignant lesions [41,44]. GPC-3 is also positive in melanoma and nonseminomatous germ cell tumors such as choriocarcinoma. Its sensitivity is known to be high (~80%), but it needs further validation [41]. 

#### 2.4.4. MOC-31

Most HCCs are negative or weakly positive for MOC-31, but it is consistently (80%–100%) expressed in cholangiocarcinoma and metastatic adenocarcinoma from colorectum, pancreas, stomach, lung, breast, and ovary [45,46]. MOC-31 is also expressed in a majority of neuroendocrine tumors, urothelial carcinomas, and renal cell carcinomas [41]. Therefore, it can help distinguish HCC from other tumors.

#### 2.4.5. α-Fetoprotein (AFP)

AFP is an oncofetal glycoprotein. Serum levels increase in patients with HCC, yolk sac tumors, hepatitis, and cirrhosis. HCC is immunoreactive for AFP in 17%–68% of paraffin embedded sections and in 45%–50% of cell blocks. Almost all hepatoid adenocarcinomas, both primary and metastatic adenocarcinomas, are reported as AFP positive [42].

## 3. Candidate Markers for Prognosis

Although the etiological factors and the natural history are well defined, HCC are quite heterogeneous and clinical behaviors are hard to predict. Therefore, it is necessary to establish robust methods capable of evaluating the prognosis of HCC patients. 

### 3.1. Chromosomal Alterations

Chromosome aberrations are frequently detected in HCC and molecular cytogenetic approaches such as comparative genomic hybridization (CGH) have provided information on copy number alterations (CNAs) in HCC. Patterns of CNAs provide useful information that can be used in heterogeneic HCC to identify genetic events involved in hepatocarcinogenesis and associated with clinical characteristics including prognosis. Among CNAs in HCC, gains of 1q, 8q, and 20q, and losses of 4q, 8p, 13q, 16q, and 17p have been frequently reported, but not necessarily with clinical or prognostic implications [47]. Kim *et al*. defined recurrently altered chromosomal regions in HCCs using whole-genome array-CGH [48]. They found that the extent of chromosomal alterations correlated with tumor grade, size and microvascular invasion. Among the genes in the recurrently gained regions on 1q, expression of *KIF14* and *TPM3* was significantly increased, suggesting their oncogenic potential in HCC. Recurrent loss in 9p24.2–p21.1 and gain in 8q11.21–q24.3 were associated with high tumor grade and microvascular invasion, respectively. Gene enrichment analysis showed that functions such as cytokine receptor binding and defense response to virus pathways are significantly enriched in high grade-related RARs [48]. Saelee *et al*. observed DNA alterations on chromosomes 5q34, 6p25.2, and 8q12.1 in Thai HCC patients and found that allelic loss on chromosome 5q34 correlated with poor survival in HCC, indicating the potential of this chromosomal region as a prognostic marker [49]. Poon *et al*. examined 158 HBV-associated HCCs by CGH analysis and classified them into three subgroups using self-organizing tree algorithm [50]. Gains in 1q21–23 and 8q22–24 were identified as genomic events associated with the early development of HCC, and the gain in 3q22–24 was associated with tumor recurrence and poor survival. Although the chromosome aberration data reported in HCC have been relatively rarer than expression or methylation profiles so far, CNA profiles, together with expression, epigenomic and clinical data, will be useful resources as a prognostic marker. 

### 3.2. DNA Methylation Profiles

Aberrant DNA methylation is frequently observed in human cancers; global DNA hypomethylation is associated with activation of protooncogenes and genomic instability; hypermethylation on CpG islands in the promoter regions of tumor suppressor genes results in transcriptional silencing and genomic instability [51,52,53,54]. It has been shown that multiple tumor-related genes, such as the *E-Cadherin* and *Hypermethylated-in-Cancer (HIC)-18* genes, are silenced by DNA hypermethylation in HCC [55,56,57]. Since DNA methylation status is altered in a coordinated manner, it is important to have a global picture of methylation changes to interpret the results correctly. However, only a few previous studies have examined genome-wide DNA methylation status in HCC.

Calvisi *et al*. analyzed the global levels of DNA methylation and the methylation status of 105 putative tumor suppressor genes and found that the extent of genome-wide hypomethylation and CpG hypermethylation correlates with clinical phenotypes and prognosis of HCC [58]. However, there was no significant association between methylation patterns and any etiologic agents in their study. They identified activation of Ras and downstream Ras effectors (*ERK, AKT*, and *RAL*) by epigenetic silencing of inhibitors of the Ras pathway in all HCC. Selective inactivation of *SPRY1* and *-2, DAB2*, and *SOCS4* and *-5* genes and inhibitors of angiogenesis (*BNIP3, BNIP3L, IGFBP3*, and *EGLN2*) was found to be associated with poor prognosis. Gao *et al*. used methylated CpG island amplification microarrays to investigate DNA methylation in cancerous and precancerous tissues from HCC [59]. They found aberrant DNA methylation of several important genes, such as *DUSP2* and *BMP6*, which are known to be associated with tumorigenesis. Early detection of aberrant DNA methylation in these genes in precancerous tissues might be useful in risk and prognosis assessment of HCC. Arai *et al*. reported that the extent of DNA methylation alterations and the degree of DNA methylation alterations increased during hepatocarcinogenesis from precancerous stage to HCC [60]. DNA methylation status was significantly different between HBV- and HCV-positive patients with HCCs in both noncancerous liver tissue and cancerous tissue, suggesting that the HBV-related carcinogenetic pathway may result in distinct DNA methylation profiles. They suggested the types of DNA methylation profiles that were able to discriminate a poor-outcome group from a favorable-outcome group. Therefore, risk estimation and prognostication of HCC based on DNA methylation profiles using liver biopsy specimens may be useful for close follow-up of patients who are at higher risk of HCC development.

### 3.3. Gene Expression Profiles

The profiling of gene expression patterns have provided new insights into the molecular pathogenesis of various human malignancies, including HCC. However, gene expression profiling is even more important to elucidate the mechanisms behind prognostic diversity and resistance against therapeutic agents. Although there have been many genome-wide gene expression studies, we focused on a couple of them which reported prognostic implications with their data. Ye *et al*. suggested a molecular signature that can classify metastatic HCC and identified genes relevant to metastasis and survival [61]. Among the identified genes, Osteopontin, which was overexpressed in metastatic HCC, was identified as a lead gene in the signature. They showed that an Osteopontin-specific antibody effectively blocked HCC cell invasion *in vitro* and inhibited pulmonary metastasis of HCC cells in nude mice. Therefore, Osteopontin can act as both a diagnostic marker and a potential therapeutic target for metastatic HCC. Several genes belonging to cell adhesion and matrix degradation were also identified to be associated with HCC metastasis, including genes encoding SPP1, α9-integrin, interleukin-2 receptor, serine proteinase inhibitor member-5, matrix metalloproteinase-9, leukocyte immunoglobulin-like receptor subfamily A member-2 and CD37 antigen. The potential of *SPP1* as a diagnostic marker was also emphasized because it can be found to be elevated in body fluids in cancer patients and as a target for therapy of HCC patients with metastatic potential. 

Lee *et al*. reported that human HCC can be subdivided into two subclasses that are associated with survival based on gene expression data [62]. It is possible that the two subclasses of HCC might represent different cellular origins, but it can be caused by considerable molecular heterogeneity within each HCC subclass. After this, Lee *et al*. reported that individuals with HCC who shared a gene expression pattern with fetal hepatoblasts had a poor prognosis by studying gene expression patterns from human HCC, mouse HCC and rat fetal hepatoblasts and adult hepatocytes [63]. It was hypothesized that HCC of this subtype may arise from hepatic progenitor cells. Analyses of gene networks showed that activation of AP-1 transcription factors might have key roles in development of this subtype. 

Iikazu *et al*. used mRNA expression profiles of tissue specimens from patients with HCC using oligonucleotide microarrays representing about 6000 genes and developed a scoring system for predicting early intrahepatic recurrence [64]. They identified 12 genes for the system, which could classify all early-stage and late-stage HCC. By combining their system with the TNM staging, they could predict early intrahepatic recurrence or non-recurrence. Among the 12 genes, *TNFAIP3* was considerably down-regulated in HCC with venous invasion. *HLADRA* and *TRIM22* were also down-regulated in HCC with early intrahepatic recurrence. Since these genes are immune-related, immunotherapy can be considered to reduce recurrence and invasion of HCC and their scoring system, based on the 12 genes, could be useful when such immunotherapy is being considered for patients with HCC.

Yamashita *et al*. reported that a hepatic stem cell marker, epithelial cell adhesion molecule (EpCAM) expression was significantly elevated in premalignant hepatic tissues and in a subset of HCC and suggested its possibility as an early biomarker of HCC [65]. EpCAM-positive HCC showed a molecular signature having features of hepatic progenitor cells, whereas EpCAM-negative HCC displayed the molecular features of mature hepatocytes. EpCAM-positive and EpCAM-negative HCC could be further subclassified into four groups with prognostic implications by combining the level of AFP. They found a close correlation between Wnt-h-catenin signaling and EpCAM-positive HCC. Wnt-h-catenin signaling is known to be activated during liver development and in HCC [66,67,68]. They have recently shown that EpCAM is a direct transcriptional target of the Wnt-h-catenin signaling pathway, further emphasizing the functional link between these two molecular nodes [69].

## 4. Conclusions

HCC exhibits numerous molecular abnormalities, which may be involved in the process of HCC development and progression. Thus, it is important to identify accurate predictors of prognosis and a reasonable selection criterion that can be applied to patients with HCC, particularly with early stage HCC, for rational treatment decisions remains a challenging task [70]. As reviewed in this article, many potentially useful molecular biomarkers have been reported, but almost none have been incorporated into the conventional TNM staging system yet. Because of the small study sizes used for most of the studies, predictors reviewed in this article are only suggestive and need to be confirmed in larger independent populations for the clinical use. In addition, since most HCC samples were obtained from hepatitis B virus–positive patients, cautions are required to apply the study results to other populations with different clinical characteristics. Given the heterogeneity of etiology and clinical behaviors of HCC, it would be very difficult to find one biochemical or molecular marker that is both specific and sensitive enough. Combination of pathological features and biomarkers with high sensitivity and specificity will be more efficient and practical for early diagnosis and prognostication of HCC.

## References

[1] Parkin D.M., Bray F., Ferlay J., Pisani P. (2001). Estimating the world cancer burden: Globocan 2000. Int. J. Cancer.

[2] Shimozawa N., Hanazaki K. (2004). Longterm prognosis after hepatic resection for small hepatocellular carcinoma. J. Am. Coll. Surg..

[3] Soresi M., Magliarisi C., Campagna P., Leto G., Bonfissuto G., Riili A., Carroccio A., Sesti R., Tripi S., Montalto G. (2003). Usefulness of alpha-fetoprotein in the diagnosis of hepatocellular carcinoma. Anticancer Res..

[4] Zhang Y.J., Wu H.C., Shen J., Ahsan H., Tsai W.Y., Yang H.I., Wang L.Y., Chen S.Y., Chen C.J., Santella R.M. (2007). Predicting hepatocellular carcinoma by detection of aberrant promoter methylation in serum DNA. Clin. Cancer Res..

[5] Wong I.H., Lo Y.M., Zhang J., Liew C.T., Ng M.H., Wong N., Lai P.B., Lau W.Y., Hjelm N.M., Johnson P.J. (1999). Detection of aberrant p16 methylation in the plasma and serum of liver cancer patients. Cancer Res..

[6] Chu H.J., Heo J., Seo S.B., Kim G.H., Kang D.H., Song G.A., Cho M., Yang U.S. (2004). Detection of aberrant p16INK4A methylation in sera of patients with liver cirrhosis and hepatocellular carcinoma. J. Korean Med. Sci..

[7] Kim J.W., Ye Q., Forgues M., Chen Y., Budhu A., Sime J., Hofseth L.J., Kaul R., Wang X.W. (2004). Cancer-associated molecular signature in the tissue samples of patients with cirrhosis. Hepatology.

[8] Craig J.R., Zakim D., Boyer T. (2003). Tumors of the liver. Hepatology: A Textbook of Liver Disease.

[9] Jia H.L., Ye Q.H., Qin L.X., Budhu A., Forgues M., Chen Y., Liu Y.K., Sun H.C., Wang L., Lu H.Z., Shen F., Tang Z.Y., Wang X.W. (2007). Gene expression profiling reveals potential biomarkers of human hepatocellular carcinoma. Clin. Cancer Res..

[10] Budhu A., Forgues M., Ye Q.H., Jia H.L., He P., Zanetti K. A., Kammula U.S., Chen Y., Qin L.X., Tang Z.Y., Wang X.W. (2006). Prediction of venous metastases, recurrence, and prognosis in hepatocellular carcinoma based on a unique immune response signature of the liver microenvironment. Cancer Cell.

[11] Khien V.V., Mao H.V., Chinh T.T., Ha P.T., Bang M.H., Lac B.V., Hop T.V., Tuan N.A., Don L.V., Taketa K., Satomura S. (2001). Clinical evaluation of lentil lectin-reactive alpha-fetoprotein-L3 in histology-proven hepatocellular carcinoma. Int. J. Biol. Markers.

[12] Yao D.F., Dong Z.Z., Yao M. (2007). Specific molecular markers in hepatocellular carcinoma. Hepatobiliary Pancreat. Dis. Int..

[13] Kamoto T., Satomura S., Yoshiki T., Okada Y., Henmi F., Nishiyama H., Kobayashi T., Terai A., Habuchi T., Ogawa O. (2002). Lectin-reactive alpha-fetoprotein (AFP-L3%) curability and prediction of clinical course after treatment of non-seminomatous germ cell tumors. Jpn. J. Clin. Oncol..

[14] Yoshida S., Kurokohchi K., Arima K., Masaki T., Hosomi N., Funaki T., Murota M., Kita Y., Watanabe S., Kuriyama S. (2002). Clinical significance of lens culinaris agglutinin-reactive fraction of serum alpha-fetoprotein in patients with hepatocellular carcinoma. Int. J. Oncol..

[15] Cillo U., Navaglia F., Vitale A., Molari A., Basso D., Bassanello M., Brolese A., Zanus G., Montin U., D'Amico F., Ciarleglio F.A., Carraro A., Bridda A., Burra P., Carraro P., Plebani M., D'Amico D.F. (2004). Clinical significance of alpha-fetoprotein mRNA in blood of patients with hepatocellular carcinoma. Clin. Chim. Acta.

[16] Yao D.F., Dong Z.Z., Yao D.B., Wu X.H., Wu W., Qiu L.W., Wang H.M., Meng X.Y. (2004). Abnormal expression of hepatoma-derived gamma-glutamyltransferase subtyping and its early alteration for carcinogenesis of hepatocytes. Hepatobiliary Pancreat. Dis. Int..

[17] Yao D.F., Huang Z.W., Chen S.Z., Huang J.F., Lu J.X., Xiao M.B., Meng X.Y. (1998). Diagnosis of hepatocellular carcinoma by quantitative detection of hepatoma-specific bands of serum gamma-glutamyltransferase. Am. J. Clin. Pathol..

[18] Pasche B. (2001). Role of transforming growth factor beta in cancer. J. Cell Physiol..

[19] Okumoto K., Hattori E., Tamura K., Kiso S., Watanabe H., Saito K., Saito T., Togashi H., Kawata S. (2004). Possible contribution of circulating transforming growth factor-beta1 to immunity and prognosis in unresectable hepatocellular carcinoma. Liver Int..

[20] Teicher B.A. (2001). Malignant cells, directors of the malignant process: role of transforming growth factor-beta. Cancer Metastasis Rev..

[21] Kim Y.J., Lee H.S., Im J.P., Min B.H., Kim H.D., Jeong J.B., Yoon J.H., Kim C.Y., Kim M.S., Kim J.Y., Jung J.H., Kim L.H., Park B.L., Shin H.D. (2003). Association of transforming growth factor-beta1 gene polymorphisms with a hepatocellular carcinoma risk in patients with chronic hepatitis B virus infection. Exp. Mol. Med..

[22] Scharf J.G., Dombrowski F., Ramadori G. (2001). The IGF axis and hepatocarcinogenesis. Mol. Pathol..

[23] Cantarini M.C., de la Monte S.M., Pang M., Tong M., D'Errico A., Trevisani F., Wands J.R. (2006). Aspartyl-asparagyl beta hydroxylase over-expression in human hepatoma is linked to activation of insulin-like growth factor and notch signaling mechanisms. Hepatology.

[24] Wang Z., Ruan Y.B., Guan Y., Liu S.H. (2003). Expression of IGF-II in early experimental hepatocellular carcinomas and its significance in early diagnosis. World J. Gastroenterol..

[25] Shiota G., Okano J., Kawasaki H., Kawamoto T., Nakamura T. (1995). Serum hepatocyte growth factor levels in liver diseases: clinical implications. Hepatology.

[26] Breuhahn K., Longerich T., Schirmacher P. (2006). Dysregulation of growth factor signaling in human hepatocellular carcinoma. Oncogene.

[27] Vejchapipat P., Tangkijvanich P., Theamboonlers A., Chongsrisawat V., Chittmittrapap S., Poovorawan Y. (2004). Association between serum hepatocyte growth factor and survival in untreated hepatocellular carcinoma. J. Gastroenterol..

[28] Yamagamim H., Moriyama M., Matsumura H., Aoki H., Shimizu T., Saito T., Kaneko M., Shioda A., Tanaka N., Arakawa Y. (2002). Serum concentrations of human hepatocyte growth factor is a useful indicator for predicting the occurrence of hepatocellular carcinomas in C-viral chronic liver diseases. Cancer.

[29] Srivastava P.K. (2005). Immunotherapy for human cancer using heat shock protein-peptide complexes. Curr. Oncol. Rep..

[30] Yao D.F., Wu X.H., Su X.Q., Yao M., Wu W., Qiu L.W., Zou L., Meng X.Y. (2006). Abnormal expression of HSP gp96 associated with HBV replication in human hepatocellular carcinoma. Hepatobiliary Pancreat. Dis. Int..

[31] Sakamoto M., Mori T., Masugi Y., Effendi K., Rie I., Du W. (2008). Candidate molecular markers for histological diagnosis of early hepatocellular carcinoma. Intervirology.

[32] Markiewski M.M., Mastellos D., Tudoran R., DeAngelis R.A., Strey C.W., Franchini S., Wetsel R.A., Erdei A., Lambris J.D. (2004). C3a and C3b activation products of the third component of complement (C3) are critical for normal liver recovery after toxic injury. J. Immunol..

[33] Lee I.N., Chen C.H., Sheu J.C., Lee H.S., Huang G.T., Chen D.S., Yu C.Y., Wen C.L., Lu F.J., Chow L.P. (2006). Identification of complement C3a as a candidate biomarker in human chronic hepatitis C and HCV-related hepatocellular carcinoma using a proteomics approach. Proteomics.

[34] Capurro M., Wanless I.R., Sherman M., Deboer G., Shi W., Miyoshi E., Filmus J. (2003). Glypican-3: a novel serum and histochemical marker for hepatocellular carcinoma. Gastroenterology.

[35] Hippo Y., Watanabe K., Watanabe A., Midorikawa Y., Yamamoto S., Ihara S., Tokita S., Iwanari H., Ito Y., Nakano K., Nezu J., Tsunoda H., Yoshino T., Ohizumi I., Tsuchiya M., Ohnishi S., Makuuchi M., Hamakubo T., Kodama T., Aburatani H. (2004). Identification of soluble NH2-terminal fragment of glypican-3 as a serological marker for early-stage hepatocellular carcinoma. Cancer Res..

[36] Capurro M.I., Xiang Y.Y., Lobe C., Filmus J. (2005). Glypican-3 promotes the growth of hepatocellular carcinoma by stimulating canonical Wnt signaling. Cancer Res..

[37] Giannelli G., Marinosci F., Trerotoli P., Volpe A., Quaranta M., Dentico P., Antonaci S. (2005). SCCA antigen combined with alpha-fetoprotein as serologic markers of HCC. Int. J. Cancer.

[38] Guido M., Roskams T., Pontisso P., Fassan M., Thung S.N., Giacomelli L., Sergio A., Farinati F., Cillo U., Rugge M. (2008). Squamous cell carcinoma antigen in human liver carcinogenesis. J. Clin. Pathol..

[39] Beneduce L., Castaldi F., Marino M., Quarta S., Ruvoletto M., Benvegnu L., Calabrese F., Gatta A., Pontisso P., Fassina G. (2005). Squamous cell carcinoma antigen-immunoglobulin M complexes as novel biomarkers for hepatocellular carcinoma. Cancer.

[40] Trerotoli P., Fransvea E., Angelotti U., Antonaci G., Lupo L., Mazzocca A., Mangia A., Antonaci S., Giannelli G. (2009). Tissue expression of Squamous Cellular Carcinoma Antigen (SCCA) is inversely correlated to tumor size in HCC. Mol. Cancer.

[41] Kakar S., Gown A.M., Goodman Z.D., Ferrell L.D. (2007). Best practices in diagnostic immunohistochemistry: hepatocellular carcinoma *versus* metastatic neoplasms. Arch. Pathol. Lab. Med..

[42] Varma V., Cohen C. (2004). Immunohistochemical and molecular markers in the diagnosis of hepatocellular carcinoma. Adv. Anat. Pathol..

[43] Lau S.K., Prakash S., Geller S.A., Alsabeh R. (2002). Comparative immunohistochemical profile of hepatocellular carcinoma, cholangiocarcinoma, and metastatic adenocarcinoma. Hum. Pathol..

[44] Yamauchi N., Watanabe A., Hishinuma M., Ohashi K., Midorikawa Y., Morishita Y., Niki T., Shibahara J., Mori M., Makuuchi M., Hippo Y., Kodama T., Iwanari H., Aburatani H., Fukayama M. (2005). The glypican 3 oncofetal protein is a promising diagnostic marker for hepatocellular carcinoma. Mod. Pathol..

[45] Morrison C., Marsh W., Frankel W.L. (2002). A comparison of CD10 to pCEA, MOC-31, and hepatocyte for the distinction of malignant tumors in the liver. Mod. Pathol..

[46] Niemann T.H., Hughes J.H., De Young B.R. (1999). MOC-31 aids in the differentiation of metastatic adenocarcinoma from hepatocellular carcinoma. Cancer.

[47] Lau S.H., Guan X.Y. (2005). Cytogenetic and molecular genetic alterations in hepatocellular carcinoma. Acta Pharmacol. Sin..

[48] Kim T.M., Yim S.H., Shin S.H., Xu H.D., Jung Y.C., Park C.K., Choi J.Y., Park W.S., Kwon M.S., Fiegler H., Carter N.P., Rhyu M.G., Chung Y.J. (2008). Clinical implication of recurrent copy number alterations in hepatocellular carcinoma and putative oncogenes in recurrent gains on 1q. Int. J. Cancer.

[49] Saelee P., Wongkham S., Bhudhisawasdi V., Sripa B., Chariyalertsak S., Petmitr S. (2008). Allelic loss on chromosome 5q34 is associated with poor prognosis in hepatocellular carcinoma. J. Cancer Res. Clin. Oncol..

[50] Poon T.C., Wong N., Lai P.B., Rattray M., Johnson P.J., Sung J.J. (2006). A tumor progression model for hepatocellular carcinoma: bioinformatic analysis of genomic data. Gastroenterology.

[51] Jones P.A., Baylin S.B. (2007). The epigenomics of cancer. Cell.

[52] Wilson A.S., Power B.E., Molloy P.L. (2007). DNA hypomethylation and human diseases. Biochim. Biophys. Acta.

[53] Herman J.G., Baylin S.B. (2003). Gene silencing in cancer in association with promoter hypermethylation. N. Engl. J. Med..

[54] Chen R.Z., Pettersson U., Beard C., Jackson-Grusby L., Jaenisch R. (1998). DNA hypomethylation leads to elevated mutation rates. Nature.

[55] Yoshiura K., Kanai Y., Ochiai A., Shimoyama Y., Sugimura T., Hirohashi S. (1995). Silencing of the E-cadherin invasion-suppressor gene by CpG methylation in human carcinomas. Proc. Natl. Acad. Sci. USA.

[56] Kanai Y., Ushijima S., Hui A.M., Ochiai A., Tsuda H., Sakamoto M., Hirohashi S. (1997). The E-cadherin gene is silenced by CpG methylation in human hepatocellular carcinomas. Int. J. Cancer.

[57] Kanai Y., Hui A.M., Sun L., Ushijima S., Sakamoto M., Tsuda H., Hirohashi S. (1999). DNA hypermethylation at the D17S5 locus and reduced HIC-1 mRNA expression are associated with hepatocarcinogenesis. Hepatology.

[58] Calvisi D.F., Ladu S., Gorden A., Farina M., Lee J.S., Conner E.A., Schroeder I., Factor V.M., Thorgeirsson S.S. (2007). Mechanistic and prognostic significance of aberrant methylation in the molecular pathogenesis of human hepatocellular carcinoma. J. Clin. Invest..

[59] Gao W., Kondo Y., Shen L., Shimizu Y., Sano T., Yamao K., Natsume A., Goto Y., Ito M., Murakami H., Osada H., Zhang J., Issa J.P., Sekido Y. (2008). Variable DNA methylation patterns associated with progression of disease in hepatocellular carcinomas. Carcinogenesis.

[60] Arai E., Ushijima S., Gotoh M., Ojima H., Kosuge T., Hosoda F., Shibata T., Kondo T., Yokoi S., Imoto I., Inazawa J., Hirohashi S., Kanai Y. (2009). Genome-wide DNA methylation profiles in liver tissue at the precancerous stage and in hepatocellular carcinoma. Int. J. Cancer.

[61] Ye Q.H., Qin L.X., Forgues M., He P., Kim J.W., Peng A.C., Simon R., Li Y., Robles A.I., Chen Y., Ma Z.C., Wu Z.Q., Ye S.L., Liu Y.K., Tang Z.Y., Wang X.W. (2003). Predicting hepatitis B virus-positive metastatic hepatocellular carcinomas using gene expression profiling and supervised machine learning. Nat. Med..

[62] Lee J.S., Chu I.S., Heo J., Calvisi D.F., Sun Z., Roskams T., Durnez A., Demetris A.J., Thorgeirsson S.S. (2004). Classification and prediction of survival in hepatocellular carcinoma by gene expression profiling. Hepatology.

[63] Lee J.S., Heo J., Libbrecht L., Chu I.S., Kaposi-Novak P., Calvisi D.F., Mikaelyan A., Roberts L.R., Demetris A.J., Sun Z., Nevens F., Roskams T., Thorgeirsson S.S. (2006). A novel prognostic subtype of human hepatocellular carcinoma derived from hepatic progenitor cells. Nat. Med..

[64] Iizuka N., Oka M., Yamada-Okabe H., Nishida M., Maeda Y., Mori N., Takao T., Tamesa T., Tangoku A., Tabuchi H., Hamada K., Nakayama H., Ishitsuka H., Miyamoto T., Hirabayashi A., Uchimura S., Hamamoto Y. (2003). Oligonucleotide microarray for prediction of early intrahepatic recurrence of hepatocellular carcinoma after curative resection. Lancet.

[65] Yamashita T., Forgues M., Wang W., Kim J.W., Ye Q., Jia H., Budhu A., Zanetti K.A., Chen Y., Qin L.X., Tang Z.Y., Wang X.W. (2008). EpCAM and alpha-fetoprotein expression defines novel prognostic subtypes of hepatocellular carcinoma. Cancer Res..

[66] Micsenyi A., Tan X., Sneddon T., Luo J.H., Michalopoulos G.K., Monga S.P. (2004). Beta-catenin is temporally regulated during normal liver development. Gastroenterology.

[67] Monga S.P., Monga H.K., Tan X., Mule K., Pediaditakis P., Michalopoulos G.K. (2003). Beta-catenin antisense studies in embryonic liver cultures: Role in proliferation, apoptosis, and lineage specification. Gastroenterology.

[68] Branda M., Wands J.R. (2006). Signal transduction cascades and hepatitis B and C related hepatocellular carcinoma. Hepatology.

[69] Yamashita T., Budhu A., Forgues M., Wang X.W. (2007). Activation of hepatic stem cell marker EpCAM by Wnt-beta-catenin signaling in hepatocellular carcinoma. Cancer Res..

[70] Budhu A., Jia H.L., Forgues M., Liu C.G., Goldstein D., Lam A., Zanetti K.A., Ye Q.H., Qin L.X., Croce C.M., Tang Z.Y., Wang X.W. (2008). Identification of metastasis-related microRNAs in hepatocellular carcinoma. Hepatology.

